# Complete Genome Sequence of Campylobacter hepaticus RBCL71delta, Associated with Spotty Liver Disease in Organic Pasture-Raised Laying Hens in Georgia, USA

**DOI:** 10.1128/mra.01309-22

**Published:** 2023-02-22

**Authors:** Roel Becerra, Julia Ienes-Lima, Jenny Nicholds, Catherine M. Logue

**Affiliations:** a Poultry Diagnostic and Research Center, Department of Population Health, College of Veterinary Medicine, University of Georgia, Athens, Georgia, USA; Indiana University, Bloomington

## Abstract

Spotty liver disease (SLD) is as an important cause of disease in egg-producing flocks. The organisms implicated include Campylobacter hepaticus and Campylobacter bilis. Here, we report the complete genome sequence of *C. hepaticus* strain RBCL71delta and three additional strains, isolated from a layer flock in Georgia, USA.

## ANNOUNCEMENT

Campylobacter hepaticus has been identified as the causative agent of spotty liver disease (SLD) in poultry (1–4). In the United States, two complete genomes of *C. hepaticus*, UF2019SK1 and USA52, are available (5, 6). Here, we report the first complete genome sequences of four *C. hepaticus* strains isolated from organic, pasture-raised laying hens (Georgia, USA). Bile samples collected from birds on the outbreak farm were plated directly on blood agar and cultured under microaerophilic conditions at 37°C for 7 days, with confirmation by PCR (7).

Whole-genome sequencing of strain RBCL71delta and three other isolates (RBCL76delta, RBCL81delta, RBCL91delta) was carried out commercially.

Genomic DNA was extracted from strains grown microaerophilically on blood agar at 37°C for 7 days using the MagAttract high-molecular-weight (HMW) DNA kit (Qiagen, Hilden, Germany) and quantified using the Qubit double-stranded DNA (dsDNA) high-sensitivity (HS) assay kit (Life Technologies, Carlsbad, CA). The DNA quality was evaluated using a NanoDrop 2000 spectrophotometer (Thermo Fisher Scientific, Waltham, MA) and analyzed on an E-Gel SizeSelect 2% agarose gel (Invitrogen, Waltham, MA). The SMRTbell Express template prep kit v2.0 (Pacific Biosciences, Menlo Park, CA) was used to prepare the genomic library, with BluePippin (Sage Science, Beverly, MA) size selection to target a library size of >7 kb. Sequencing was performed on the PacBio Sequel II platform (Pacific Biosciences) to generate circular consensus sequencing (CCS) reads.

Sequencing yielded 317,377 reads totaling 300,011,149 bp (coverage, 200×), and their quality was examined using FastQC v0.11.9 (8). The reads were assembled using Canu v2.1.1 (9) with the “pacbio-hifi” option. Circlator v1.5.5 (10) was used to trim and circularize the contigs and Pilon v1.20 (11) to polish them, resulting in single chromosomes. The circularized genomes were rotated to the default starting gene, *dnaA*. Annotation was conducted using the NCBI Prokaryotic Annotation Pipeline and RAST v2.0 (12). Virulence genes were identified using the Virulence Factor Database (VFDB) (13). Unless otherwise noted, default parameters were used for all software tools. Statistics for all four genomes, including *N*_50_ values and GC contents, are shown in Table 1.

**TABLE 1:** Characteristics of genome assemblies and annotation statistics

Parameter	Data for strain:			
	RBCL71delta	RBCL76delta	RBCL81delta	RBCL91delta
Total no. of reads (bp)	317,377	240,714	339,305	333,083
Total assembly length (bp)	1,516,079	1,560,236	1,564,828	1,550,659
No. of contigs	1	4	3	2
GC content (%)	28.03	28.07	28.06	28.04
*N*_50_ (bp)	1,516,079	1,516,087	1,516,087	1,487,452
No. of subsystems	293	292	294	294
No. of coding sequences	1,488	1,527	1,535	1,517
No. of RNAs (tRNAs, rRNAs)	54	55	60	56
Avg depth of sequencing coverage (×)	200	200	200	200
GenBank accession no.	CP104325	JAOBQM000000000	JAOBQL000000000	JAOBQK00000000
SRA accession no.	SRR21491726	SRR21504727	SRR21504726	SRR21504725
BioProject accession no.	PRJNA878518	PRJNA878793	PRJNA878793	PRJNA878793

Of the four genomes, only one was closed into a single chromosome (RBCL71delta). *C. hepaticus* RBCL71delta has a GC content of 28.03% and comprises 1,516,079 bp, with 1,488 predicted protein-coding sequences (CDS) encoding 54 RNAs (9 rRNA genes, 42 tRNAs, 3 noncoding RNAs [ncRNAs]). Additionally, it encodes 101 virulence genes, including *cadF*, the *kps* operon, and *ciaB*, responsible for adherence, capsule biosynthesis, and invasion, respectively. Furthermore, the chromosome encodes genes related to colicin V production and the multidrug resistance efflux pump CmeABC. No plasmids were identified in any of the genomes.

### Data availability.

The genome sequence of *C. hepaticus* RBCL71delta was deposited in GenBank under the accession numbers in Table 1.
